# Real-time Imaging of Rabies Virus Entry into Living Vero cells

**DOI:** 10.1038/srep11753

**Published:** 2015-07-07

**Authors:** Haijiao Xu, Xian Hao, Shaowen Wang, Zhiyong Wang, Mingjun Cai, Junguang Jiang, Qiwei Qin, Maolin Zhang, Hongda Wang

**Affiliations:** 1 State Key Laboratory of Electroanalytical Chemistry, Changchun Institute of Applied Chemistry, Chinese Academy of Sciences, Changchun, Jilin, P.R. China; 2 Key Laboratory of Tropical Marine Bio-resources and Ecology, South China Sea Institute of Oceanology, Chinese Academy of Sciences, Guangzhou, P.R. China; 3 University of Chinese Academy of Sciences, Beijing, P.R. China; 4 Key Laboratory of Zoonosis, Ministry of Education Institute of Zoonosis, Jilin Universiy, Changchun, Jilin, P.R. China

## Abstract

Understanding the mechanism of rabies virus (RABV) infection is vital for prevention and therapy of virulent rabies. However, the infection mechanism remains largely uncharacterized due to the limited methods and viral models. Herein, we utilized a powerful single-virus tracking technique to dynamically and globally visualize the infection process of the live attenuated rabies vaccine strain-SRV_9_ in living Vero cells. Firstly, it was found that the actin-enriched filopodia is in favor of virus reaching to the cell body. Furthermore, by carrying out drug perturbation experiments, we confirmed that RABV internalization into Vero cells proceeds via classical dynamin-dependent clathrin-mediated endocytosis with requirement for intact actin, but caveolae-dependent endocytosis is not involved. Then, our real-time imaging results unambiguously uncover the characteristics of viral internalization and cellular transport dynamics. In addition, our results directly and quantitatively reveal that the intracellular motility of internalized RABV particles is largely microtubule-dependent. Collectively, our work is crucial for understanding the initial steps of RABV infection, and elucidating the mechanisms of post-infection. Significantly, the results provide profound insight into development of novel and effective antiviral targets.

The rabies is an acute zoonotic disease with highly fatality, causing in excess of 50 000 human deaths annually, the majority of which occur in Asia^1^. As the culprit of the rabies, rabies virus (RABV) belonging to the genus Lyssavirus of the Rhabdoviridae family, is highly neurotropic^2^,^3^. RABV is characterized as an enveloped, nonsegmented, negative single stranded RNA virus encoding five proteins, N, P, M, G and L^4^. This nucleocapsid of rabies virus is surrounded in “bullet” by a lipid bilayer containing the transmembrane glycoprotein G, which is exposed on the virus surface as a trimeric spike^5^,^6^. The RABV G plays a critical role in the infectious cycle^7^,^8^,^9^. The human health has been severely threatened by rabies virus for a long time. To design antiviral drug, it is urgent to deeply investigate the cellular mechanisms underlying the viral infection.

The viral infection was determined as a complex process, which involves in the virus-cell attachment, the viral entry, the membrane fusion and the intracellular transport from the cytosol entry site to gene replication site^10^,^11^,^12^,^13^,^14^,^15^. Inherently, the viral infection process is a very intriguing virus-cell interaction, in which viruses try their best to take advantage of host cell machineries for efficient infection. The first step of viral infection is viral attachment to cell surface. Remarkably, after attachment, viruses have been frequently observed to exploit dynamic cell surface protrusions-filopodia to gain access into the entry sites at the cell body^16^,^17^,^18^,^19^. Most animal viruses make use of receptor-mediated endocytosis to enter their host cells. The endocytic pathway includes various types, of which clathrin-mediated endocytosis (CME) and caveolae-dependent endocytosis (CavME) are leading entry pathways^20^. Subsequently, to overcome the barrier from the complex cellular environment, the internalized viruses have adapted to utilize the cellular transport systems to reach a specific site for genome release^21^. In this process, many viruses have been reported to exploit the microtubules as transport tracks, such as adenovirus, herpes simplex virus, HIV, and influenza virus^10^,^15^,^22^,^23^.

Understanding the mechanism of virus infection is vital for therapy of viral diseases. It has been established that rabies virus enter the host cell via receptor-mediated endocytosis and subsequent low pH-dependent fusion^24^,^25^, and the endocytic pathway is proven to be clathrin-mediated^26^. However, these previous experimental methods were mainly based on electron microscopy (EM), which cannot disclose the dynamic process of viral infection with the disadvantage to only acquire static images. The appearance of real-time fluorescence imaging techniques solves this problem. In recent years, it has been applied to the investigation of RABV infection mechanism including the entry pathway and intracellular transport^27^,^28^. Moreover, live cell imaging technique has indicated that RABV exploits axonal transport mechanisms during CNS invasion by interacting with p75NTR^29^. However, the life cycle of rabies virus is very sophisticated and many questions about RABV infection mechanism remain unknown^30^. Therefore, it is imperative to develop a novel viral model to combine with the excellent method to further investigate the infectious mechanism.

In this study, the live attenuated rabies vaccine strain-SRV_9_ was used as the novel viral model to investigate the infection mechanism of RABV. Compared to recombinant RABV, apart from the advantage of production convenience, the live and integral rabies strain with all the characteristics of RABV morphologically and compositionally, is a good alternative to further investigate the infection mechanism of authentic RABV. Significantly, we employed real-time tracking technique to monitor individual SRV_9_ particles in living cells, which allows us to intuitively visualize dynamic interactions between viruses and host cells. The combination of the novel model with the excellent method facilitates us to further characterize the authentic RABV infection mechanism. Our results globally and directly revealed the infection process of RABV exploited by a single virus including virus-cell attachment, internalization and intracellular trafficking, which not only support the previous results from recombinant viruses, but also provide some novel findings.

## Results and Discussion

### Biological characterization of purified SRV_9_

**Figure 1:**
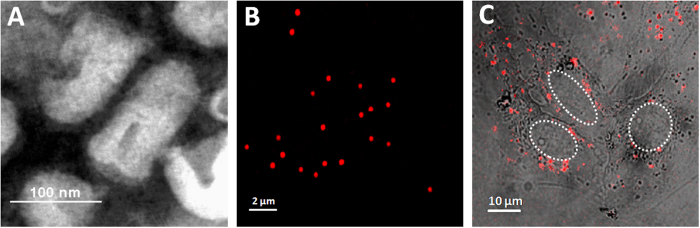
Biological characterization of purified SRV_9_. (**A**) Electron micrograph of density gradient-purified SRV_9_ negatively stained with PTA. (**B**) Confocal image of Cy5-stained SRV_9_ particles immobilized on glass coverslips. (**C**) The fluorescence image of Vero cells that internalized Cy5-labled SRV_9_ particles. The nucleus region was circled by white dashed line.

The SRV_9_ belongs to living attenuated rabies vaccine strain with good safety and immunogenicity, resulting from the alteration of Arg→Ser in the 333 position of glycoprotein of rabies viruses^31^. The amino acid mutation does not alter the features of rabies viruses in morphology. The electron microscopic analysis showed that the SRV_9_ particles are in bullet shape and measured on average 180nm in length and 80nm in width (Fig. 1A). The SRV_9_ is morphologically consistent with the wild type (WT) RABV. To carry out single particle fluorescence imaging, we covalently labeled the glycoprotein of SRV_9_ with fluorescent dye molecules Cy5-NHS. The Cy5 is an amino-active dye that spontaneously binds with the glycoproteins, and its surface density was sufficiently high so that its fluorescence allowed the labeled SRV_9_ to be clearly detected. The confocal laser scanning microscopy (CLSM) was used to image the Cy5-labeled SRV_9_ particles adsorbed onto glass coverslips. As shown in Fig. 1B, some scattered individual SRV_9_ particles were observed, which indicates that the purified SRV_9_ particles were suitable for single particle tracking experiment. Next, we co-incubated the cells with labeled SRV_9_ particles for 30 min at 37 °C. The confocal image revealed that numerous virus particles had entered the cells and distributed throughout the interior of the cell, including the cell periphery and perinuclear positions (Fig. 1C). The result indicated that the labeled SRV_9_ still enable to enter the cell.

To study whether the dye-labeling has effect on the infectivity of viruses, we carried out the immunofluorescence assay and Western blotting analysis (Figure S1 and Figure S2 in the Supplementary Information). The results suggested that the dye-labeling has no significant effect on the infectivity of viruses. These experimental results suggest that we have successfully purified relatively homogeneous populations of SRV_9_ particles. The fluorescence labeled SRV_9_ particles are well appropriate for single-particle tracking and imaging. In this work, we will choose the SRV_9_ as a new model to dissect rabies virus infection mechanisms.

### SRV_9_ are associated with filopodia prior to viral entry

Generally, virus-cell interaction starts with the attachment of the virus particle to the cell surface. After the attachment, the virus reaches to endocytic zones at the cell body to complete the internalization. It has been reported that the member of Rhabdoviruses VSV was directly transported into clathrin-coated pits by filopodia after the cell attachment^18^. The filopodia are actin-enriched cell surface protrusions and considered as the feeler of the cell to probe the extracellular environment. However, whether filopodia actively contribute to SRV_9_ entry is still unclear. Herein, we applied live-cell fluorescence imaging to investigate the process of viral entry.

To better perform the fluorescence imaging experiment, a labeling strategy was taken, in which the Vero cells and SRV_9_ were labeled with the lipophilic membrane dye DiO and Cy5-NHS, respectively. In this process, the filopodia as cell surface protrusion were also stained by DiO.

**Figure 2:**
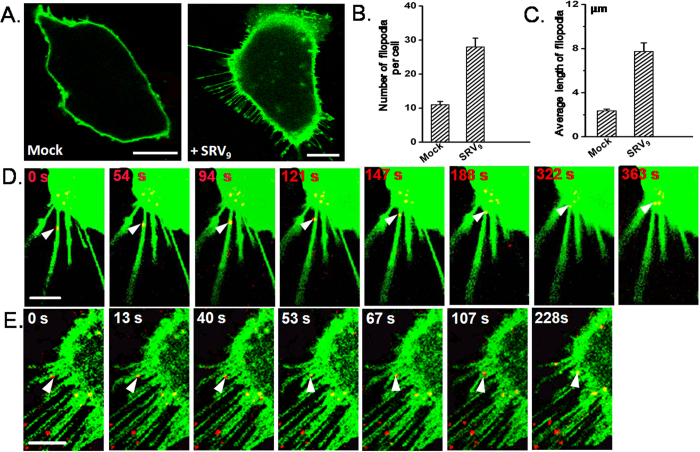
The correlation of the viruses with filopodia. (**A**) The confocal images of Vero cells (green) that unexposed or exposed to SRV_9_. (**B**) The number of filopodia per cell with or without SRV_9_ exposure was quantified. Error bars represent standard error of the mean (SEM). (**C**) The average length of filopodia with or without SRV_9_ exposure was quantified. Error bars represent SEM. In D and E, SRV_9_ (red) were added to Vero cells (green) and immediately monitored over time by confocal fluorescence microscopy. (**D**) Arrowheads mark the movement of single virus particles *via* retrograde transport from the filopodium periphery toward the cell body. (**E**) An example of single virus particles reaching the cell body through filopodial retraction where the filopodial tip is marked by arrowheads. Scale bars: 10 μm.

To observe the association of SRV_9_ with the filopodia, we implemented the following contrasting experiment. Visualization of the Vero cells in unexposed SRV_9_ revealed few filopodia (Fig. 2A; mock). Notably, after cells exposured to SRV_9_ for short periods of time, a strong induction of filopodia was observed (Fig. 2A; +SRV_9_). Simultaneously, we quantitatively analyzed the effect of the SRV_9_ exposure on the number of filopodia per cell and the average length of filopodia in more than 100 cells. (Fig. 2B,C). The results indicated SRV_9_ addition has activated more filopodia production and elongation, which may induce increased virus uptake from the surrounding environment.

Next, we further investigated whether filopodia structures play a role in viral entry. Time-lapse confocal microscopy was performed on live Vero cells infected by SRV_9_. We observed retrograde viral transport of an individual viral particle (i.e., “surfing”) along filopodia until reaching the cell body (Fig. 2D, see Movie S1 in the Supplementary Information). In addition to the viral active transport, another mode of viral particle getting to the cell body via filopodia is also observed, which is termed as filopodial retraction. As observed (Fig. 2E, see Movie S2 in the Supplementary Information), the individual viral particle attached to the filopodia was transferred to cell body as the filopodia retracted. Meanwhile, another filopodia served as the “helper” filopodia in the homing process of the viral particle towards the cell body. The similar phenomenon has been found in other studies^32^.

Our results indicate that SRV_9_ are closely related to the filopodia in viral entry. This is in accordance with the results of previous studies^19^,^33^. Meanwhile, the coincidence with the behavior of VSV suggests that the Rhabdoviridae viruses possibly share the same mechanism in the process of viral reaching to the cell body. Movement along filopodia may represent an efficient infectious pathway, by which viruses can rapidly and directly reach the endocytic zones for entry into cells. However, the activation mechanism of filopodia needs further investigation.

### Entry of SRV_9_ into Vero cells is via clathrin-mediated and actin-dependent pathway rather than caveola-dependent

**Figure 3:**
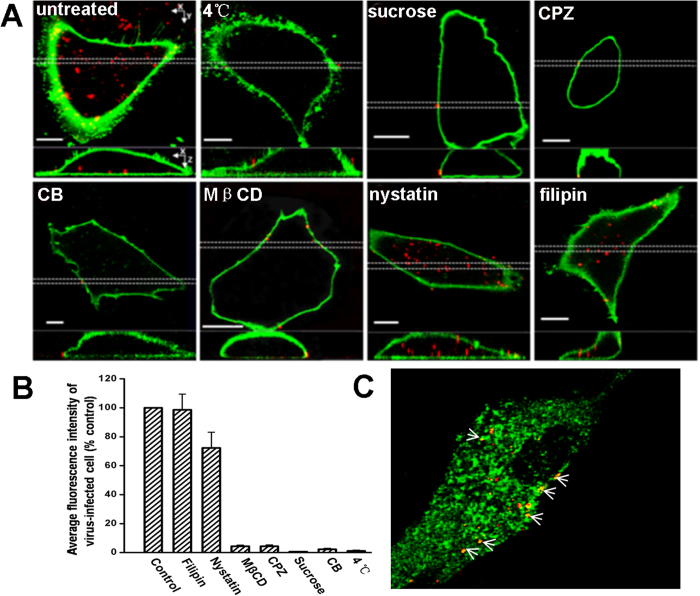
SRV_9_ Entry depends on clathrin-mediated endocytosis rather than caveola-dependent. The Vero cells were labeled by DiO (green). In the control group, the Vero cells were incubated with Cy5-labeled SRV_9_ for 30 min at 37 °C and washed by PBS immediately before imaging. In treated group, the Vero cells were pretreated by low temperature, as well as several inhibitors including sucrose (200 mM), CPZ (10μg/ml), CB (20 μM), MβCD (10 mM), nystatin (100 μM), and filipin (10 μg/ml) for 30 min prior to SRV_9_ infection, respectively. More than 100 cells were examined by CLSM in each treatment. (**A**) Three-dimensional confocal images of Vero cells infected with SRV_9_ under different treatment conditions. Successive z-stacks spaced by 200 nm were recorded to construct the 3D image. The XY image plane is located about 4 μm above the bottom of the cell. The XZ image plane that used for recording in all experiments is approximately 1;μm thick as indicated by the white dashed lines. Scale bars: 10 μm. (**B**) Quantitation of viral entry rate. The viral entry rate was quantified as the average fluorescence intensity of virus-infected cell relative to that for control cells. The viral entry rate of control cells was factitiously set as 100%. Error bars represent SEM. (**C**) SRV_9_ particles colocalize with clathrin. Vero cells were transfected with pEGFP-LCa prior to incubation with Cy5-labeled SRV_9_. The arrows indicate an individual SRV_9_ particle (red) colocalized with clathrin (green). Scale bars are 10 μm.

Although the entry mechanism of RABV has been reported, the reconstructed RABV were used in previous studies^8^,^27^. Here, we employed live SRV_9_ as the surrogate of wild-type RABV to comprehensively evaluate the endocytosis pathway. Several disruption drugs that specifically hinder different entry pathways were utilized. In the inhibition experiments, cells were pretreated with various inhibitors prior to SRV_9_ infection. The inhibitors were maintained throughout the experiment process. As positive control, the Vero cells are infected by SRV_9_ without any drug pretreatment. As shown in Fig. 3A, a large number of virions were seen inside the untreated control cell.

First, to verify whether or not the SRV_9_ enters the cell via endocytosis pathway, we incubated Vero cells with SRV_9_ at 4 °C for 30 min. Compared to the control cells, the low temperature pretreatment made the SRV_9_ internalization drastically inhibited (>98%) (Fig. 3A,B), suggesting the endocytosis pathway was exploited. Then, the role of clathrin-mediated pathway in SRV_9_ entry was assessed by using sucrose and CPZ (Chlorpromazine). Hypertonic sucrose is generally considered as an inhibitor of CME by causing dissociation of clathrin vesicles from the plasma membrane^34^, while CPZ inhibition on CME is attributable to blocking the assembly of clathrin-coated pits^35^. Compared to the control, pretreatment of Vero cells with sucrose (200 mM) or CPZ (10 μg/mL) strongly inhibited SRV_9_ internalization, about 99% and 95%, respectively (Fig. 3A,B). Dynamin is an essential cellular GTPase involved in clathrin-dependent and lipid raft/caveola-dependent endocytic pathways^36^. To validate the role of dynamin in SRV_9_ entry, we treated the Vero cells using dynasore, which rapidly and specifically inhibits the GTPase activity of dynamin^37^. SRV_9_ particles were obviously reduced in infected dynasore-treated cells (Figures S3 in the Supplementary Information). Furthermore, to further determine if SRV_9_ particles were associated with clathrin, Vero cell were transfected with pEGFP-LCa and infected with SRV_9_. As shown in Fig. 3C, some virus particles were observed to colocalize with clathrin clusters (green) as indicated by white arrows. The proportion of colocalization is statistically analyzed at about 58.52% when virus infecting for 30min. Meanwhile, the dynamic process of single virus particle entry into cell through the clathrin is observed using real-time tracking technique (Movie S3 in Supplementary Information). Together, these results indicate that dynamin-dependent and clathrin-mediated endocytosis is involved SRV_9_ entry.

Recently, some studies have revealed that the rhabdoviruses VSV, IHNV, and ABLV are internalized through clathrin-dependent pathways with the help of actin^38^,^39^,^40^. The contribution of actin to SRV_9_ entry was studied using the actin-depolymerizing drug CB (cytochalasin B). As expected, the entry of SRV_9_ was seriously inhibited by the pretreatment of cells with cytochalasin B (>97%) (Fig. 3A,B), suggesting that clathrin-mediated SRV_9_ internalization is associated with actin polymerization. Meanwhile, the effect of CB inhibition on viral entry also may be correlated with inhibition of actin-enriched filopodia.

As reported previously, some viruses have exploited more than one type of endocytic pathway to efficiently enter host cells^41^,^42^,^43^,^44^. Accordingly, we determined whether or not the other pathway is involved in SRV_9_ entry into host cells. MβCD (methyl-β-cyclodextrin) is generally used to extract cholesterol from the membranes. When the Vero cells were pretreated with MβCD, the uptake of SRV_9_ was apparently reduced by around 95% (Fig. 3A,B). Although the caveolae formation is strictly dependent on cholesterol^45^, the cholesterol depletion by MβCD also inhibits clathrin-coated pit (CCP) budding^46^. Consequently, the result merely shows that the uptake of SRV_9_ was dependent of membrane cholesterol. This finding is compatible with a clathrin- or caveolae- mediated entry pathway for SRV_9_.

To further test the contribution of CavME to SRV_9_, the effect of the cholesterol-binding drug nystatin or filipin was studied. Due to the caveolae invagination requiring for cholesterol, isolation with sterol-binding drugs nystatin or filipin will dramatically block caveola-dependent endocytosis. However, pretreatment of Vero cells with nystatin or filipin almost did not obviously decrease the uptake of SRV_9_ into Vero cells (25% and 2%, respectively) (Fig. 3A,B). These results suggest that SRV_9_ entry into Vero cells is caveolae-independent.

Taken together, our results intuitively disclosed that SRV_9_ was internalized into Vero cells by a clathrin-dependent and actin-dependent pathway similar with VSV, IHNV and ABLV. Besides, the CavME is proven to not contribute to SRV_9_ entry into Vero cells. Through overall analysis of live SRV_9_ entry pathway, our results reflect the entry pathway of WT rabies virus to the maximum. These data presented here further reveal that the rhabdoviruses, such as VSV, IHNV, ABLV, RABV, may exploit the same endocytosis mechanism^38^,^40^,^42^.

### Single-virus tracking of SRV_9_ entry and its intracellular transport

**Figure 4:**
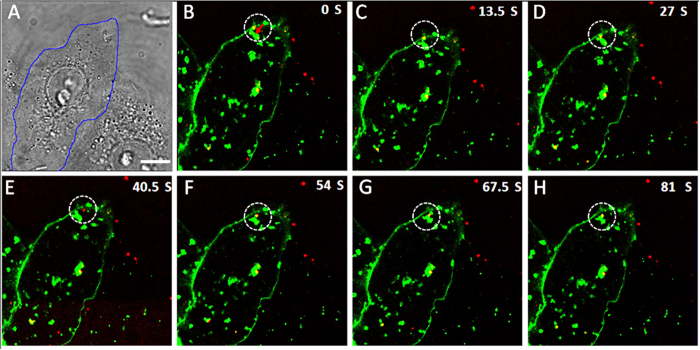
The process of individual SRV_9_ entry into Vero cell. (**A**) Bright-field image of a Vero cell as circled by blue line. (**B–H**) Selected frames reveal the process that SRV_9_ particle was internalized into the Vero cell. In this experiment, Vero cells labeled with DiO (green) were infected with Cy5-labeled SRV_9_ (red). The imaging was performed at 10 min after the infection. The white dashed circles show the site of SRV_9_ entry into the Vero cell. Scale bar: 10 μm.

To investigate the infection process of individual SRV_9_, we observed the internalization and the transport of SRV_9_ by tracking single virus in live cells using time-lapse confocal laser scanning microscope. The SRV_9_ and Vero cell membranes were labeled with Cy5-NHS and DiO, respectively. The cellular vesicles were also nonspecifically stained by the lipophilic dye DiO. Firstly, the viruses were added to Vero cells for 10 min at 37 °C, then tracked in real-time. Figure 4A represents the image of Vero cells in bright field. The border of the Vero cell corresponding to that in the fluorescence images is marked by blue line. It is easy to identify the sites of SRV_9_ entry through visualizing the fusion site of SRV_9_ and the Vero cell membrane. The time-lapse confocal microscope image showed that the red fluorescences (Cy5-labeled SRV_9_) were colocalized with the green fluorescences (DiO-labeled cell membrane) as circled by white dashed line in Fig. 4B. The colocalization indicated that the virus was attached to cell membrane. After attachment, the virus continued to interact with the cell membrane for a short time (Fig. 4B,C). The interaction promoted the cell membrane to invaginate to form vesicle, as shown in Fig. 4D, then the virus was wrapped in the vesicle. At this moment, the vesicle was still located at cell membranes. The preexisting vesicle (pointed by red arrow in Fig. 4B) bound to the new-forming vesicle, which provided tensile force to facilitate the vesicles to detach from the membrane. In Fig. 4E, the single virus had already escaped from the vesicle and entered the cell. Afterwards, the virus fused with the preexisting vesicle and moved towards the cytoplasm. The whole internalization process of SRV_9_ was recorded in Movie S4 (Supplementary Information).

**Figure 5:**
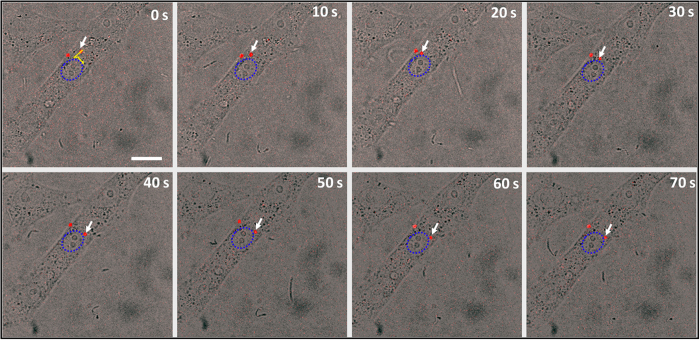
The process of individual SRV_9_ transport in a Vero cell. Vero cells were infected with SRV_9_ (red), and imaged 10 min after the infection. The nucleus region was circled by blue dashed line. The white arrow points a single virus inside the cell. The trajectory of a Cy5-labeled virus inside a cell is shown by yellow line. The movement of the virus was recorded by real-time confocal microscopy. The time interval of serial images was 10 s. Scale bar: 20 μm.

Next, to further reveal the infection process, we utilized the real-time tracking to monitor the individual virus transport in live cells. As shown in Fig. 5, a single virus was imaged in the cell as pointed by white arrow, and the cell nucleus was circled by the blue dashed line. The typical trajectory of a virus starting in the cell periphery, moving to the perinuclear region and finally locating there is shown in Fig. 5. The rapid movement process is recorded in Movie S5 in Supplementary Information. In short, the single-virus tracking is an excellent tool to follow the fate of individual virus particle in live cells^47^. By means of this technique, for the first time, we directly revealed characteristics of the internalization and transport behavior of single SRV_9_ particle in living cells, which is significant for understanding the initial stage of viral infection process.

### Rapid microtubule-dependent Motility of SRV_9_

Upon internalization, virus usually exploits cellular transport system to deliver their genomes to specific compartments for replication. To observe the movement of SRV_9_ in cells in detail, we infected DiO-labeled Vero cells with Cy5-labeled SRV_9_. Then, the time-lapse individual particle imaging was performed in living cells using confocal microscope. The imaging was carried out about 30 minutes after virus infection. At this moment, the great majority of particles had entered cells and exhibited high motility inside the cells.

**Figure 6:**
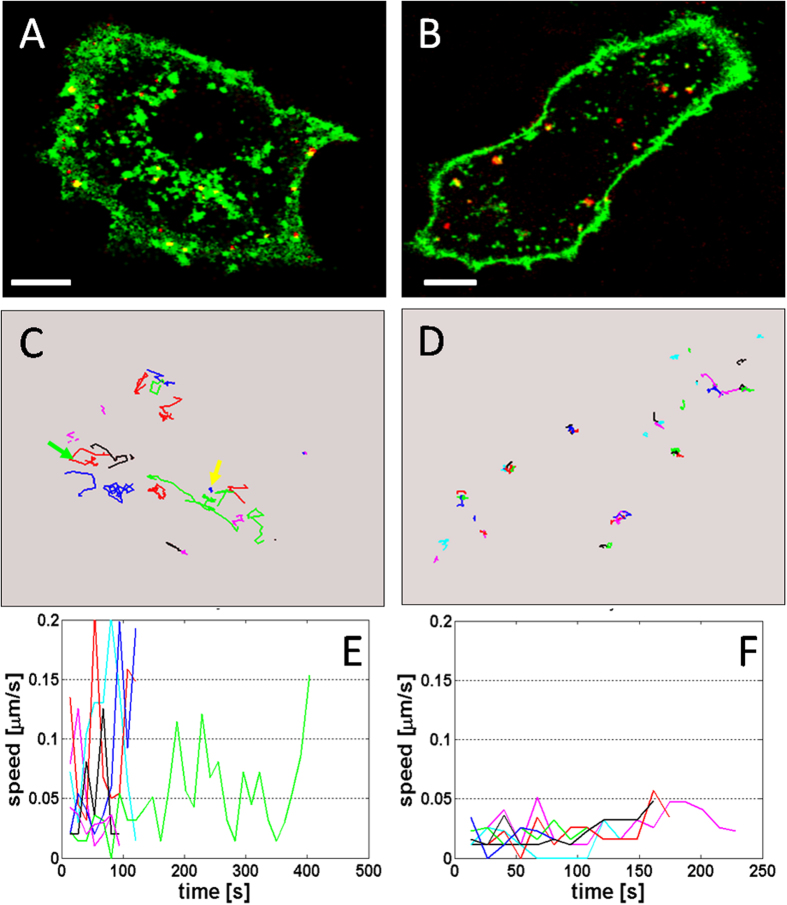
Motility of SRV_**9**_ in the untreated, nocodazole-treated Vero cells. (**A**,**B**) Confocal images of SRV_9_ (red) in live cells labeled with DiO dye (green) under untreated and nocodazole-treated conditions, respectively. (**C**, **D**) Representative trajectories corresponding to the untreated and nocodazole-treated images, respectively. Their instantaneous speeds as a function of time are shown in E and F. The green arrow and the yellow arrow in (**C**) indicate relatively high transport speed and low transport speed in a limited region, respectively. Scale bar: 10 μm.

Figure 6A and Movie S6 in Supplementary Information represent typical examples of SRV_9_ motility in Vero cells. The Matlab software allowed us to analyze the trajectory of single-virus particle inside the cell, with the ability to distinguish the virions inside or on the cell from outside the cell. The analytical results were displayed in Fig. 6C, showing various types of movements. Most of viruses particles were able to achieve relatively high transport speeds (marked by the green arrow in Fig. 6C), despite some particles showed comparatively low speed motion (marked by the yellow arrow in Fig. 6C). The SRV_9_ particles were able to achieve speeds nearly up to 0.2 μm s^−1^ (blue trajectory velocity profile in Fig. 6E). Some of these relatively rapid moving particles displayed highly directed movements. It is noted that the slowly moving particles were localized to vesicular structures nonspecifically stained by the lipophilic dye DiO. However, the relatively rapid moving particles didn’t colocalized with the vesicular structures, which means that these viruses were possibly fused with specific endocytic vesicles that need to be labeled by specific makers or were free inside the cytosol. It is worth noting that the actual speeds are virtually much higher than the speeds calculated from the two-dimensional projection. The reason accounting for the deviation is that the virus particles moved inside the cell typically in all three dimensions, but only a two- dimensional projection of three-dimensional motion was measured.

As a fundamental component of cytoskeleton, microtubule is usually essential for the intracellular transport of many viruses. To validate whether SRV_9_ motility in cell was the microtubule-dependent, the effect of microtubule-disrupting drug was evaluated. In this experiment, cells were pretreated with nocodazole prior to the incubation with SRV_9_. By contrast with the control, the treatment of cells with nocodazole that can depolymerize microtubules resulted in great reduction of the viral motility. A large number of SRV_9_ trajectories inside the cell were analyzed (Fig. 6B), showing that the majority of SRV_9_ particles were almost static (Fig. 6D, Movie S7 in Supplementary Information). The trajectory velocity profile in Fig. 6F also revealed that the transport speed was extremely low. This result initially indicated that the microtubules may be involved in the intracellular transport of SRV_9_.

**Figure 7:**
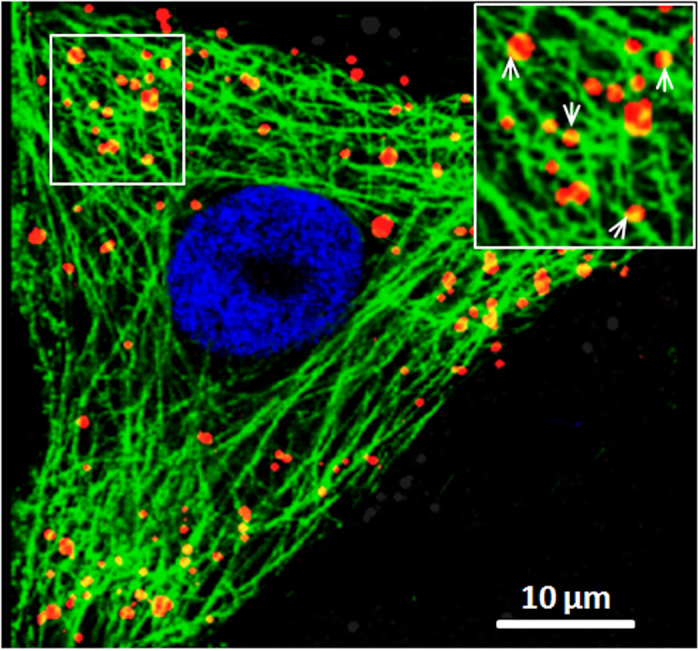
Colocalization of SRV_9_ particles with microtubules. Immunofluorescence was performed at cells infected by Cy5-labele SRV_9_ (red) after 30 min using anti-tublin antibody (green). The white square region is magnified and shown in the inset. The white arrows represent some examples of colocalization of Cy5-labeled SRV_9_ with FITC-tagged microtubules. Scale bar: 10 μm.

Next, we performed the colocalization experiment to analyze the involvement of microtubules in the intracellular motility of SRV_9_ (Fig. 7). The microtubules were recognized by antibody against tubulin. In the magnified image (inset), these yellow signals as indicated by white arrows represented the overlap of the microtubules (green) and Cy5-labeled SRV_9_ (red), indicating that some SRV_9_ colocalized with the microtubules. Our statistical result indicated that about 77.6% of SRV_9_ virions are colocalized with microtubules at 30 min for virus infection. In addition, SRV_9_ particles were observed to move along microtubules during infection (see Movie S8 in the Supplementary Information). The results suggested that microtubules may be crucial for virus transport during infection.

**Figure 8:**
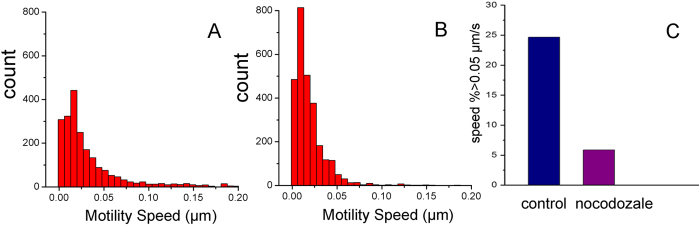
(**A**,**B**) The histogram of motility speed distribution of SRV_9_ in the untreated and nocodazole-treated Vero cells, respectively. (**C**) Comparison of SRV_9_ motility with the speed >0.05 μm s^−1^ between the untreated and nocodazole-treated Vero cells.

Because of complexity and heterogeneity of the intracellular environment, the viruses can exploit more than one transport system simultaneously to efficiently infect their hosts^48^,^49^. Therefore, it is necessary to characterize and quantitatively analyze the viral motility by tracking a large number of virus particles in cells. The statistical analyses were implemented on tens of thousands of trajectories in many independent experiments. The Fig. 8A,B presented statistics histogram of speed in untreated cell and nocodazole-treated cell, respectively. The statistical analysis from the two group cells is shown in Fig. 8C, displaying that the percentage of trajectories with peak speeds >0.05 μm s^−1^ is 24.7% in untreated cells, and 5.9% in nocodazole-treated cells. The 4.2-fold reduction in the speed of SRV_9_ motility occurred in microtubule-depolymerized cells. Therefore, we speculated that SRV_9_ motility was closely related to the microtubules in living cells. Recently, the microtubule-mediated behavior of virus motility has been proven as a complex event^50^. Future work will be required to determine whether this microtubule-associated motility way ultimately contributes to productive infection, and if so what underlying mechanism is involved.

Due to high fatality of RABV, there have been extensive studies on RABV infection mechanism. It is a pity that the thorough understanding of the infection mechanism is limited by methods and models. Compared to previous studies, in this work, we utilized the real-time single-virus tracking method to study the infection mechanism of the live attenuated rabies vaccine strain-SRV_9_. The real-time tracking represents an excellent system that allowed us to gain a detailed observation of the infection process of single-virus particle, while the live attenuated rabies vaccine strain-SRV_9_ as a novel model enables to further reflect the infection characteristics of the wide-type RABV.

In summary, we combined the advanced imaging technique with a novel viral model to globally investigate the infection mechanism of RABV. The data presented in this study suggest that RABV internalization into host cells occurs through a dynamin-dependent CME pathway, rather than caveolae-dependent. Furthermore, the results showed that the actin cytoskeleton plays an indispensable role in efficient virus entry. The intact actin is necessary for the clathrin-mediated uptake of RABV, meanwhile, the actin-enriched filopodia are significant for viral attachment to the cell body. Our results revealed that the RABV may have the same endocytosis mechanism as other members of the Rhabdoviruses, such as VSV, IHNV and ABLV. Additionally, the real-time imaging results displayed the characteristics of viral internalization and cellular transport dynamics, and revealed that the viral cellular transport is largely microtubule- dependent. Our work is not only significant for deeply understanding the mechanism of viral infection, but also paves the way for the development of novel and effective antiviral targets.

## Methods

### Cell and virus

Vero cells (from Shanghai Institute of Biological sciences) were maintained in a 5% CO_2_ environment at 37 °C in minimum Eagle medium (MEM, GIBCO) with 10% FBS (GIBCO), penicillin (100 internationalunits mL^−1^), and streptomycin (100 μg mL^−1^), and passaged every 2–3 days. SRV_9_ were amplified in Vero cells. The titer of virus is detected by sandwich ELISA. The cultures with high content of viruses were collected. One part were freezed at −70 °C as reserve, and the other were prepared for purification. The cultures were concentrated by using Zn (AC)_2_, then purified in linear 10% ~ 50% sucrose gradients. Concentrated viruses stocks were stored in PBS containing 10 mM HEPES (pH 7.4) at −80 °C.

### Fluorescence Labeling of SRV_9_

Amine-reactive Cy5 (OPE TECH) was solubilized in DMSO at the concentration of 1mg/mL. To label SRV_9_, Cy5 was incubated with purified viruses at a final concentration of 1 μg/mL in a carbonate buffer (pH 9.3) at room temperature for 2 h with gentle vortexing. To remove the unbound Cy5, the virus mixture was centrifuged at 17000 g, finally eluted in PBS (pH 7.4) and stored at −80 °C. The labeled viral aggregates were immediately removed with 0.2 μm pore size filters before experiments and examined under electron microscopy or fluorescence microscope.

### Drug Treatment

To perform fluorescence imaging, Vero cells were cultured in MEM with 10% FBS for 2 or 3 days to achieve 75% confluence in 35-mm glass-bottomed culture dishes (Shengyou Biotechnology Co., Ltd). The cells were pretreated with various chemical inhibitors purchased from Sigma. The pretreatment of cells with nocodazole (60 μm) or cytochalasin B (20 μm) was for 50 min, respectively; while cells were preincubated with sucrose (450 mM), chlorpromazine (CPZ, 10 μg/mL), methyl-β-cyclodextrin (MβCD 10 mM), Filipin III (10 μg/mL), Nystatin (100 μm), respectively, for 30 min. The sucrose and CPZ were used to inhibit clathrin-mediated endocytosis; filipin III and nystatin were used to inhibit caveola-mediated endocytosis; MβCD was used to inhibit cholesterol content-associated endocytic pathway. The drugs were maintained in the cell culture throughout the experiments. After drug treatment, the cells were incubated with the viruses at 37 °C in a CO_2_ incubator for 30 min before experiments. Cells were washed with PBS for 3 times, then incubated in serum-free and phenol red-free MEM as prepared for experiments.

### Plasmids and Transfection

Two plasmids that respectively encodes GFP -α- tubulin, eGFP - LCa (clathrin light chain molecules) were used to transfect the Vero cells in this study. After cells were plated for 24 h, the transfection was performed by using Lipofectamine 2000 (Invitrogen) according to the manufacturer’s instructions. The cells were subsequently cultured for 24 ~ 48 h prior to imaging acquisition.

### Immunofluorescence microscopy

Cells were fixed with 4% paraformaldehyde, permeabilized with 0.2% Triton X-100 for 15 min, and blocked with bovine serum albumin (BSA) (Sigma). Cells were then washed three times with PBS and incubated with primary antibodies against tubulin (1:100) diluted in 0.2% BSA at room temperature for 2 h. After washing, FITC-conjugated goat anti-mouse IgG was added and left at room temperature for 1h. Then, the cells were incubated with Hoechst 33342 at room temperature for 5 min after washing. Cells were imaged using confocal microscopy.

### Immobilization of virions on glass coverslips

The purified and Cy5-labeled virions were immobilized as described as follows. In brief, the virions were loaded onto glass coverslips and adsorbed for 2 h at room temperature in the dark. After adsorbed onto the glass surface, the virions were directly analyzed by confocal laser scanning microscopy.

### Electron microscopy

The purified virus particles were deposited onto carbon-coated copper grids and stained with 2% phosphotungstic acid (PTA) in H_2_O (pH 7.5). Then, the virus particles were imaged using a Tecnai G2 Spirit BioTWIN transmission electron microscope (FEI, Hillsboro, OR).

### Fluorescence Microscopy and Image Analysis

The live cell fluorescence imaging was carried out using a Leica TCS SP2 confocal Microscope. Cy5-labeled SRV_9_ particles were excited with a 633 nm helium–neon laser, while the DiO-labeled membrane, GFP-tagged tubulin, and eGFP-LCa were all excited with a 488 nm Ar–Kr laser. The fluorescence images were obtained with a NA = 1.40 100×oilimmersion objective. Experiments were conducted at 37 °C unless otherwise mentioned. Data were collected using a photomultiplier tube (PMT) and processed with Leica TCS software. For real-time tracking, we utilized the series scanning imaging of Leica TCS to obtain a sequence of digital images at discrete time points. The serial images are converted to a movie using Image-Pro Plus 6.0. Imaging analysis referring to the motility of viruses is carried out using custom-written MATLAB based on an algorithm described previously^47^. The fluorescence intensity is analyzed using MATLAB.

## Additional Information

**How to cite this article**: Xu, H. *et al.* Real-time Imaging of Rabies Virus Entry into Living Vero cells. *Sci. Rep.* **5**, 11753; doi: 10.1038/srep11753 (2015).

## Supplementary Material

Supplementary Movie S1

Supplementary Movie S2

Supplementary Movie S3

Supplementary Movie S4

Supplementary Movie S5

Supplementary Movie S6

Supplementary Movie S7

Supplementary Movie S8

Supplementary Information
