# Supplementary material for: Real-time Imaging of Rabies Virus Entry into Living Vero cells
Source: Sci Rep. 2015 Jul 7;5:11753. doi: 10.1038/srep11753 (PMC4493577; doi:10.1038/srep11753)
Supplement: Supplementary Information [file srep11753-s9.pdf]

# Supplementary Information

# Real-time Imaging of Rabies Virus Entry into Living Vero cells

Haijiao Xu[1], Xian Hao[1], Shaowen Wang[2,3], Zhiyong Wang[1], Mingjun Cai[1], Junguang Jiang[1], Qiwei Qin[2,3], Maolin Zhang[4]* and Hongda Wang[1,3]*

[1] State Key Laboratory of Electroanalytical Chemistry, Changchun Institute of Applied Chemistry, Chinese Academy of Sciences, Changchun, Jilin, P.R. China, [2] Key Laboratory of Tropical Marine Bio-resources and Ecology, South China Sea Institute of Oceanology, Chinese Academy of Sciences, Guangzhou, P.R. China, [3] University of Chinese Academy of Sciences, Beijing, P.R. China, [4] Key Laboratory of Zoonosis, Ministry of Education Institute of Zoonosis, Jilin Universiy, Changchun, Jilin, P.R. China

**Corresponding author**:
*E-mail: hdwang@ciac.ac.cn; zhrei98@163.com

## 1. Immunofluorescence microscopy

To verify the dye-labeling is positive for viruses, we carried out the immunofluorescence experiment. Cells were incubated with $SRV_9$ for 48h, then and washed three times with PBS to remove the redundant medium. Subsequently, cells were fixed with 4% paraformaldehyde, permeabilized with 0.2% Triton X-100 for 15 min, and blocked with bovine serum albumin (BSA) (Sigma). Cells were then washed three times with PBS and incubated with FITC Anti-Rabies Monoclonal Globulin against viral N proteins diluted in 0.2% PBS at 37℃ for 40 min. After washing, the cells were incubated with Hoechst 33342 at room temperature for 5 min. Cells were sealed using antifade Mounting Medium, then imaged using confocal microscopy. Compared to the control cell, the fluorescence signaling was not obviously reduced in Cy5-labeled $SRV_9$ infected cell.

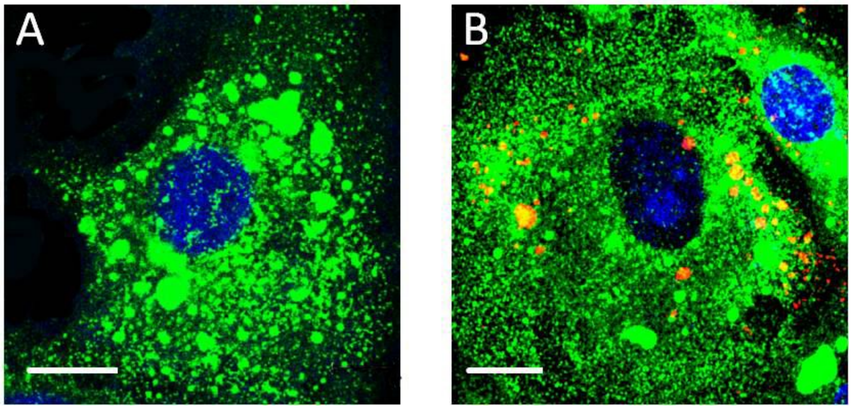


**Figure.S1.** Detection of N proteins expression in unlabeled-$SRV_9$ infected or Cy5-labeled -$SRV_9$ cells by immunostaining. (A) The cells were infected by unlabeled-$SRV_9$. (B) The cells were infected by Cy5-labeled-$SRV_9$ (Red). The infected cells were then analyzed at 48 hpi by immunofluorescence assay using anti- N proteins antibody (green). Scale bars: 10 μ m.

## 2. Western blotting

To further study the effect of the dye-labeling on the infectivity of viruses, we detected the viral protein synthesis by Western blotting analysis. The cells were infected with viruses, collected at 48 hpi and then lysed in PBS containing1xsodium dodecyl sulfate (SDS) loading buffer. Cell lysates were fractionated by SDS-polyacrylamide gel electrophoresis and transferred to polyvinylidene fluoride membranes for 70 min. After blocking with 5% (w/v) nonfat milk and washing in Phosphate-buffered saline-Tween solution, membranes were incubated with anti-P protein as primary antibody, washed and then incubated with horseradish peroxidase–goat antimouse antibody as secondary antibody. Immunoblots were visualized using an enhanced chemiluminescence detection system according to the manufacturer's protocol (Amersham ECL Western blotting detection kit). The Western blotting result shows that the yields of viral P proteins were not obviously affected in cells which were infected with Cy5-labeled $SRV_9$, compared to the unlabeled $SRV_9$.

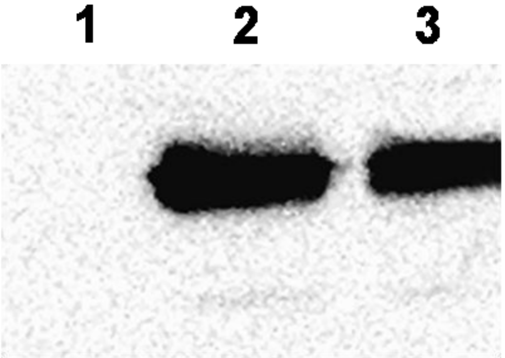


**Figure.S2.** The detection of viral P proteins expression by Western blotting. Lane 1: P expression was detected in mock-infected cells. Lane 2: P expression was detected in cells infected with unlabeled $SRV_9$. Lane 3: P expression was detected in cells infected with Cy5-labeled $SRV_9$.

## 3. The effect of dynasore on the $SRV_9$ entry into Vero cells

To study whether dynasore will affect the $SRV_9$ entry into cells, the cells are pretreated with dynasore (10 μM) for 30 min prior to virus infection. The Vero cells were labeled with DiO (green), and the $SRV_9$ virions were labeled with Cy5-NHS (red). The single cell confocal image showed in the Figure S3 is representative. More than 90% of cells showed the similar effect, and ~100 cells were observed in the treatment. The results demonstrate that the dynamin-dependent CME is involved in $SRV_9$ entry into Vero cells.

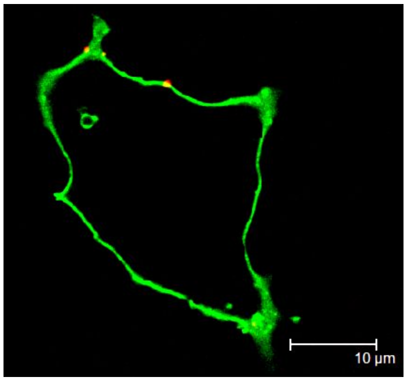


**Figure.S3.** The confocal image of Vero cell infected with $SRV_9$ under dynasore treatment condition. Scale bar is 10μm.

**Movie S1.**
$SRV_9$ moves along filopodia *via* retrograde transport towards the cell body. Movie from the time-sequence images shows individual Cy5-labeled $SRV_9$ (red) surfing along protrusion on DiO-labeled Vero cells (green), reaching the cell body. Acquisition of images occurred at 13. 4 seconds per frame.

**Movie S2.**
Retraction of filopodia serves as another means of transferring $SRV_9$ to the cell body. Movie from a series of confocal images shows that an individual $SRV_9$ particle (red) moved with the gradually-shortening filopodia (green) to arrive at the cell body. Acquisition of images occurred at 13. 4 seconds per frame.

**Movie S3.**
The internalization process of single $SRV_9$ particle into Vero cell via clathrin-coat pits. The $SRV_9$ were labeled with Cy5-NHS (red) and Vero cells (green) were transfected with pEGFP-LCa prior to incubation with Cy5-labeled $SRV_9$. Acquisition of images occurred at 13. 4 seconds per frame.

**Movie S4.**
The process of individual $SRV_9$ entry into Vero cells (The movie corresponds to Fig.4). The $SRV_9$ were labeled with Cy5-NHS (red) and Vero cells were labeled with DiO (green). Acquisition of images occurred at 13. 4 seconds per frame.

**Movie S5.**
The process of individual $SRV_9$ transport in Vero cell (The movie corresponds to Fig.5). The $SRV_9$ were labeled with Cy5-NHS (red); the Vero cells were infected with $SRV_9$ (red) and imaged 10 min after the infection. Acquisition of images occurred at 10 seconds per frame.

**Movie S6.**
The motility of $SRV_9$ in Vero cell without drug treatment (The movie corresponds to Fig.6A). The $SRV_9$ were labeled with Cy5-NHS (red) and Vero cells were labeled with DiO (green). Acquisition of images occurred at 13. 4 seconds per frame.

**Movie S7.**
The motility of $SRV_9$ in Vero cells that were pretreated with 60 μM nocodazole for 50 min in order to depolymerize microtubules (The movie corresponds to Fig.6B). The $SRV_9$ were labeled with Cy5-NHS (red) and Vero cells were labeled with DiO (green). Acquisition of images occurred at 13. 4 seconds per frame.

**Movie S8.**
The transport of $SRV_9$ particles along microtubule in the Vero cell. The Vero cells were transiently transfected with green fluorescent protein (GFP)-tagged á-tubulin (green) prior to infection. After 24 h, Cy5-labeled $SRV_9$ particles (red) were added to cells, and the images were captured immediately. Acquisition of images occurred at 13. 4 seconds per frame.
